# The Bone Strain Index: An Innovative Dual X-ray Absorptiometry Bone Strength Index and Its Helpfulness in Clinical Medicine

**DOI:** 10.3390/jcm11092284

**Published:** 2022-04-20

**Authors:** Fabio Massimo Ulivieri, Luca Rinaudo

**Affiliations:** 1 Centro per la Diagnosi e la Terapia dell’Osteoporosi, Casa di Cura La Madonnina, Via Quadronno 29, 20122 Milan, Italy; 2 Tecnologie Avanzate T.A. Srl, Lungo Dora Voghera 36, 10153 Torino, Italy; l3rinaudo@gmail.com

**Keywords:** DXA, BMD, TBS, Bone Strain Index

## Abstract

Bone strain Index (BSI) is an innovative index of bone strength that provides information about skeletal resistance to loads not considered by existing indexes (Bone Mineral Density, BMD. Trabecular Bone Score, TBS. Hip Structural Analysis, HSA. Hip Axis Length, HAL), and, thus, improves the predictability of fragility fractures in osteoporotic patients. This improved predictability of fracture facilitates the possibility of timely intervention with appropriate therapies to reduce the risk of fracture. The development of the index was the result of combining clinical, radiographical and construction-engineering skills. In fact, from a physical point of view, primary and secondary osteoporosis, leading to bone fracture, are determined by an impairment of the physical properties of bone strength: density, internal structure, deformation and fatigue. Dual X-ray absorptiometry (DXA) is the gold standard for assessing bone properties, and it allows measurement of the BMD, which is reduced mainly in primary osteoporosis, the structural texture TBS, which can be particularly degraded in secondary osteoporosis, and the bone geometry (HSA, HAL). The authors recently conceived and developed a new bone deformation index named Bone Strain Index (BSI) that assesses the resistance of bone to loads. If the skeletal structure is equated to engineering construction, these three indexes are all considered to determine the load resistance of the construct. In particular, BSI allows clinicians to detect critical information that BMD and TBS cannot explain, and this information is essential for an accurate definition of a patient’s fracture risk. The literature demonstrates that both lumbar and femoral BSI discriminate fractured osteoporotic people, that they predict the first fragility fracture, and further fragility fractures, monitor anabolic treatment efficacy and detect patients affected by secondary osteoporosis. BSI is a new diagnostic tool that offers a unique perspective to clinical medicine to identify patients affected by primary and, specially, secondary osteoporosis. This literature review illustrates BSI’s state of the art and its ratio in clinical medicine.

## 1. Introduction

Metabolic Bone Diseases, and particularly primary and secondary osteoporosis, are characterised by bone derangement that leads to fragility fractures which reduce quality of life and may cause death, primarily in the case of hip fracture [1]. Prevalence and incidence of osteoporosis are increasing worldwide, mainly because of the ageing, and osteoporotic fractures are associated with substantial social, economic, and healthcare burdens [2]. As osteoporosis is an asymptomatic or pauci-symptomatic disease, it is essential for clinicians to formulate an early diagnosis of bone derangement. This allows prescribing in time the appropriate measures to prevent fragility fractures and to monitor the efficacy of the pharmacological treatments. 

Assessment of bone status relies mainly on the Dual X-ray Absorptiometry (DXA), the World Health Organization’s gold standard diagnostic technique for the measurement of bone quantity (expressed as BMD), bone geometry (HSA, HAL), and bone quality (TBS) [3,4,5]. The instrumental diagnosis of osteoporosis is established when BMD, in terms of standard deviation from a healthy young population, is ≤ −2.5 for women in post-menopause or for men over 50, whereas a T-score ≤ −1.0 is classified as osteopenia. For men under 50 and for premenopausal women, BMD is indicated as a standard deviation from age- and sex-matched population with the cutoff set at ≤−2.0 [6]. BMD measurement is a well-known indicator of bone strength, and it has been widely used for many decades to classify patients in clinical practice.

However, it is also known that low BMD accounts for about 70% of the fragility fractures observed in the clinical practice. Furthermore, it is also well known that there is an overlap between BMD distribution of patients with and without osteoporotic fractures [7,8]. All this indicates that other elements may play a role in bone strength, like texture, geometry, deformation capability, fatigue and physical determinants of the strength of all materials, bone included [9]. These are fundamental aspects in those diseases where BMD is not significantly reduced. Still, fragility fractures are frequent, as usually observed in secondary osteoporosis due, for example, to glucocorticoids and rheumatological disorders [10], not by chance, included among the highly critical risk factors for osteoporosis in the fracture risk charts like the FRAX tool. Bone involvement is well-known in these pathologies: systemic bone loss is one of the most common comorbidities. It starts early in the disease development, even before clinical acknowledgment, as in rheumatological diseases [11]. The skeletal sites affected are mainly those with prevalent trabecular bone, like lumbar spine, but cortical bone, like femoral neck and distal radius, may also be affected [12,13], with significantly lower BMD values related to the disease duration and regardless of treatment [14]. A reduction in BMD also characterises periarticular local bone loss in RA [15,16], which seems to be associated with the development of aggressive systemic diseases [17]. Glucocorticoids (GCs) are often prescribed at a higher dose to treat secondary osteoporosis and their detrimental effect on the bone, with increased risk of fragility fracture, has long been documented in the literature [18,19].

TBS, an indirect DXA bone texture index, used since 2008, is an effective bone quality index, explaining fracture events in patients with a higher BMD receiving GC [18,20]. Nevertheless, in other diseases, like endocrinological and nephrological ones, bone quality derangement is more relevant than bone quantity loss [21]. Diabetes, for example, is an in vivo model of this particular condition where TBS is a better fracture predictor than BMD [22]. Moreover, in hyperparathyroidism [23] and chronic kidney disease [24] TBS has proven to be clinically useful [10]. TBS is an essential tool to investigate bone quality status. Still, not all necessary information to evaluate the resistance of bone to loads is taken into consideration, and there is a lack in geometry parameters and load performance. In addition, TBS is inferred from the lumbar spine scan and does not provide data about femoral bone quality status.

Bone geometry parameters, inferred from a DXA hip scan, perform as another index of bone derangement in primary and secondary osteoporosis [25,26,27]. Hip Structural Analysis (HSA) provides a mechanical description of three femoral areas of interest (Narrow Neck, Intertrochanteric and Femur Shaft) employing different parameters, like the cross-sectional area (CSA), the cross-sectional moment of inertia (CSMI), the section modulus (Z) and the buckling ratio (BR) [5]. Studies have revealed that HSA results predict hip fracture occurrence [28,29]. However, their use in patient’s management is still limited by difficulties in interpreting the structural parameters and by insufficient evidence from clinical practice settings regarding fracture prediction [5]. For this reason, scientific guidelines do not recommend its routine clinical use to assess hip fracture risk [5,30]. DXA images can automatically obtain two other geometric parameters: the neck-shaft angle (NSA) and hip axis length (HAL). Several studies have found a positive association between longer HAL and hip fracture, and it thus seems that this geometric parameter plays an important role in predicting hip fracture regardless of BMD values [5]. It is not yet clear whether the NSA may be used as an additional fracture risk parameter [5].

Undoubtedly BMD, TBS, and Hip geometry are all helpful to assess bone status in secondary osteoporosis. However, information about bone resistance to load is incomplete and data relating to deformation and fatigue are missing from a constructive point of view. One of the most complete approaches to investigating bone from a mechanical point of view is Finite Element Analysis, which allows determining the stress and strain status of an object made up of a specific material and subject to specific load conditions. This method is extensively used in engineering and has been applied successfully both in fracture risk prediction [31] and in prosthetic implants simulation [32,33].

Recently, the authors have conceived and developed an innovative DXA-derived index, calculated with Finite Element Analysis (FEA) on a greyscale of the distribution of density measured on both spine and femoral scans, namely the Bone Strain Index (BSI) [9]. BSI calculation considers information on density distribution, bone geometry and resistance to loadings on local areas. It diverges from bone mineral density (BMD) and trabecular bone score (TBS), which are based on quantifying bone mass and its distribution over the scanned area. In addition to bone density and its distribution, BSI also includes data concerning the shape of the skeletal investigated site and in-specific-conditions load applied to the bone by means of the patient’s weight. BSI is a new horizon for the evaluation of bone assessment. It offers helpful information to better understand bone quality derangement in metabolic bone diseases, particularly in secondary osteoporosis. This systematic review illustrates the state of the art of BSI and its ratio in clinical medicine. This synthesis allows clinicians to quickly and easily take note of the information that the BSI can provide for optimal management of the osteoporotic patient. The authors have checked PubMed and Scopus and searched the following expressions: bone strain index, strength index of bone and their acronyms, osteoporosis and secondary osteoporosis.

### 1.1. BSI: Its Helpfulness

Bone can be considered a complex entity, built with particular structural properties and geometrical characteristics to fulfill its natural support function: resistance to compressive, torsional and flexural loads. From a construction point of view, many factors of the skeleton have to be taken into account to explain bone strength [34] and their analysis is essential to improve our capacity to predict a structural failure. In a structure under external load, magnitude and distribution of internal stresses depend on the loading configuration, the geometry of the system and the properties of the employed materials. To avoid permanent damage and fracture, stresses and strains must remain below a specific yield-point level. Bone is subjected to the same mechanical rules and its resistance is governed by its density, geometry, internal trabecular structure and cortical thickness, all of which can be inferred from radiological images. The measurement of these parameters can be based on volumetric images (e.g., computed tomography) or planar images, where traditional radiography (X-ray) and DXA are the most common technologies. X-ray images can be analysed using different methods [34,35,36], ranging from classic beam models, usually applied to long bones [35,37,38], to the application of more complex models, such as the finite element model (FEM) [39]. The FEM method consists of dividing an object into simpler elements, to which the laws of classical mechanics apply. Forces and constraints, applied to the bone in specific regions, generate internal stresses and strains, which depend on the magnitude and the type of the solicitation, the bone geometry and the stiffness of each simple element in which the bone has been divided. Although many FEMs have been developed to investigate the bone status and fracture risk, none of them is used in routine clinical practice. Indeed, the FEM programs, to date, have not been entirely automated or adapted to clinical reporting. Furthermore, it is important to applying this method to the usually scanned femoral and lumbar anatomical sites, when employing DXA.

In recent years many studies have focused on FEM analysis of the proximal femur to estimate femoral strength and assess hip fracture risk [34,40]. However, only a few studies have dealt with the lumbar anatomical site, having demonstrated a better vertebral strength prediction of FEMs compared to a real BMD measured with DXA [41].

Recently, the authors have proposed a new DXA bone parameter, named BSI, based on lumbar and femur scan FEM analysis to improve fracture risk prediction, considering all features involved in bone strength [42,43]. The FEM analysis is conducted automatically by placing forces and constraints on a triangular mesh derived from bone segmentation performed on DXA software. For the lumbar site, each vertebra is loaded on the upper surface and constrained to the lower, as indicated in the scheme used by Colombo et al. [42]. Material properties of each triangle of the model are assigned following the experimental relations described by Morgan et al. at the lumbar site [44], whereas the force applied to the upper plate of the vertebra is calculated using the patient-specific model described in the study by Han et al. [45]. In the femoral area, the BSI algorithm is based on a condition of lateral fall, with constraints placed both on the head and the lower part of the shaft and with a subject-specific impact force (linked to the weight of the person) applied to the greater trochanter [46].

**Figure 1:**
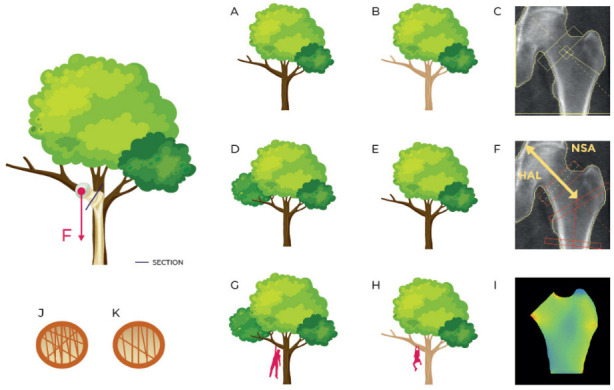
Simplification of the strain on the femur in a standing position or while walking comparing it with a tree with a force F acting on a branch. (**A**,**B**) describe the same branch with different material density (as inferred by BMD DXA scan represented in (**C**)); (**D**,**E**) describe the branches of the same material with different shape (as described by geometry parameters HSA and HAL in (**F**)); (**G**,**H**) describe different branches in different load situations (femurs with different BMD, different geometry and different applied load as represented in femoral BSI analysis in (**I**)); (**J**,**K**) describe different inner structures (concept similar to TBS even though TBS doesn’t apply to femur region).

Since the BSI value is related to its resilience to withstand an applied load, it reflects bone strength. As explained in the introduction, mechanical resistance to fracture should consider different variables: stiffness, texture, geometry, deformation capability and fatigue. In Figure 1, for example, the stress of the femur during walking is compared to that of a tree branch on which a downward force is acting. By extension, with bone densitometry, two equally shaped trees made of different types of wood represent the same situation of two femurs having different BMD. On the other hand, branches with different sizes and shapes represent the case of two femurs with different geometry, that in DXA field can be described by HSA parameter, HAL and NSA. All these parameters combine to describe the status of a bone, individually and separately. Conversely, BSI describes the stress/strain situation of a specific bone, with a specific geometry and a specifically applied load and, looking at the previous example, can describe two different branches, with different wood (BMD), shape (bone geometry), and applied load (patient’s weight).

### 1.2. Experimental Data

Experimental data on BSI were obtained from ex vivo porcine studies and works’ evidences are summarized in Table 1.

Colombo et al. demonstrated a good estimation of calculated yield strain when compared to experimental yield measured on samples of porcine vertebra (R^2^_adj_ = 0.814) [42]. The proposed algorithm was then modified considering the thickness of the model, proportional to the average width of the vertebra and to the elastic modulus attributed to each element, based on the Morgan et al. study [44]. The statistical analysis showed how the BSI values presented a higher correlation to mechanical bone strength than BMD or TBS parameters.

However, the reality is much more complex and, in some cases, differs from simple mechanical test conditions. A fracture can occur by applying an impulse load or by applying a load repeated over time (even when the stress level is below the resistance threshold in quasistatic conditions, as in the case of fatigue).

**Figure 2:**
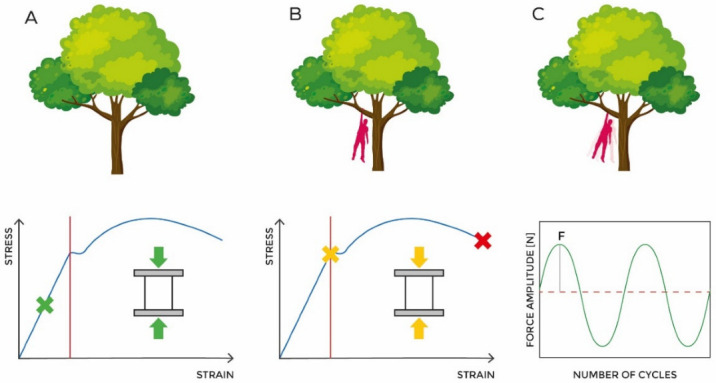
Simplification of the strain on the femur during walking with different loading conditions. (**A**) describes the normal state of a branch of a tree bearing just its weight. This situation is comparable to a femur under physiological stress. (**B**) represents a branch with a person hanging on it and can be compared to a femur that has to bear an overweight person. (**C**) represents a branch with a swinging person that repeatedly applies a force F.

Using the same comparison example between a femur and a tree branch, in Figure 2, it is possible to understand the impact of different loads applied to a structure. Indeed, probably fracture risk is different under certain physiological conditions (stress/strain within the linear elastic acceptable range), in the case of overload (stress/strain near or beyond the mechanical limit described by yield point), or again in the case of repeated stress.

Even if the simulation under repeated stress conditions is more complex, bone damage processes are involved in most of non-traumatic fracture events.

In a recent paper, Buccino et al. [47] describe how strain data can be used as an indicator of fatigue life, being directly related to progressive damage in bones. Strain values show a trend that increases with the number of cycles and with accumulated bone damage. The study underlines how the same algorithm used by BSI in the bone strain map is able to represent local strain concentrations, despite all the limitations of a bi-dimensional model and the fact that, in in vivo conditions, fatigue and progressive damage are mitigated by bone remodeling.

### 1.3. Reproducibility Data

BMD reproducibility is usually the reference standard for DXA-based measurements and this has been taken into account in all studies regarding DXA parameters. BSI in vitro and in vivo precision studies was assessed according to ISCD guidelines [6,30]. Table 2 shows an overview of BMD and BSI in vitro and in vivo precision data.

Messina et al. conducted a lumbar spine phantom study at different speed modes and soft tissue thicknesses, in order to simulate abdominal fat. The best reproducibility was found using a low-speed acquisition with 1 cm of superimposed soft tissue, whereas the worst one was a low-speed mode with 6 cm of superimposed soft tissue. BSI reproducibility slightly decreased with soft tissue thickness increase. The least significant BSI change was about three times that of BMD in all modalities and fat thicknesses [48].

Messina et al. also conducted an in vivo study of lumbar spine BSI reproducibility with 30 postmenopausal women divided into three groups, according to body mass index (BMI) and into two groups according to waist circumference (WC). BSI reproducibility decreased proportionally to BMI and WC increase, and in all cases was lower than that of BMD. The reduction of BSI reproducibility was more evident in overweight patients with BMI ≥ 30 kg/m^2^ and WC > 88 cm, as expected, since BSI is a weight sensible parameter as mentioned in the introduction [49]. Moreover, this pattern can also be explained by the noise generated by the superimposed soft tissue, which contributes to reducing X-ray image quality and accuracy [50].

Messina et al. also investigated short-term reproducibility of femoral BSI in vitro, on a phantom, and in vivo (30 subjects). Both in vitro and in vivo, BSI reproducibility was better over the total femur than at the femoral neck (95.31% versus 91.48% in vitro. 89.22% versus 88.46% in vivo). BSI in vivo reproducibility was about three times lower than BMD, confirming previous results of lumbar spine BSI [51].

## 2. Clinical Evidence

### 2.1. Primary Osteoporosis

Clinical studies since 2018 have investigated the usefulness of BSI in identifying osteoporotic patient subgroups having a particular tendency to fragility fractures [52] and in predicting successive vertebral fragility fractures [53,54]. Table 3 and Table 4 summarize all these studies regarding primary and secondary osteoporosis.

In a retrospective study, Ulivieri et al. by means of artificial neural network analyses (ANNs), studied 125 consecutive women in post-menopause, evaluating DXA parameters, biochemical markers of bone turnover and clinical data. Fracture risk was found to be low when carboxy-terminal cross-linking telopeptide of type I collagen level was low. In contrast, a positive Romberg test, associated with weakened bone strength (high lumbar BSI), appeared to be closely linked to vertebral fragility fractures, pointing the way to fragility fracture in postmenopausal women [52].

Ulivieri et al. investigated the BSI capability to predict a vertebral re-fracture in a preliminary study with 143 consecutive, fractured patients with primary osteoporosis (121 females) who had undergone an X-ray to calculate spine deformity index (SDI) and a DXA to measure BMD, TBS and BSI. Re-fracture was considered a one-unit increase in SDI. For each unit increase of BSI the hazard ratio of re-fracture, 95% confidence interval, p-value, and proportionality test *p*-value were 1.201, 0.982–1.468, 0.074, and 0.218; while for lumbar BMD 0.231, 0.028–1.877, 0.170, and 0.305; and for TBS 0.034, 0.001–2.579, 0.126, and 0.518, respectively. BSI proved to be the most accurate predictive index of re-fracture, being nearest to statistical significance [53].

This finding was confirmed by Messina et al. in a multicentric validation study conducted on 234 consecutive, fractured patients (209 females), who performed a spine X-ray to calculate SDI and DXA densitometry for BMD, TBS and BSI at the basal time and in the follow-up. For each unit increase of the investigated indexes, the univariate hazard ratio of re-fracture, 95% CI, p-value and proportionality test *p*-value are for age 1.040, 1.017–1.064, 0.0007 and 0.2529, and for BSI 1.372, 1.038–1.813, 0.0261 and 0.5179, respectively. BSI remained in the final multivariate model as a statistically significant, independent predictor of a subsequent re-fracture (1.332, 1.013–1.752 and 0.0399) together with age (1.039, 1.016–1.064 and 0.0009); for this multivariate model proportionality test, the *p*-value was 0.4604 [54].

This finding has also been settled by Sornay-Rendu et al. in a recent work on OFELY study that prospectively investigated fracture prediction of lumbar and femur BSI, demonstrating that spine and femur BSI predict incident fragility fracture in postmenopausal women, independently of FRAX values. In 261 premenopausal women, Neck and Total Hip BSI were slightly negatively correlated with age (r −0.13 and −0.15 respectively, *p* = 0.03), whereas all BSIs were correlated positively with BMI (r = 0.20 to 0.37, *p* < 0.01) and negatively with BMD (r = −0.69 to −0.37, *p* < 0.0001). In 585 postmenopausal women, Neck and Total Hip BSI were positively correlated with age (r = 0.26 and +0.31 respectively, *p* < 0.0001), whereas Spine BSI was positively correlated with BMI (r = 0.22, *p* < 0.0001) and all BSIs were negatively correlated with BMD (r = −0.81 to −0.60, *p* < 0.0001). In a median 9.3 years of follow-up, 133 postmenopausal women communicated an incident fragility fracture. These women included those with a major osteoporotic fracture and with a clinical vertebral fracture. Each SD increase in BSI value was related to a significant increase in the risk of all fragility fractures with an age-adjusted HR of 1.23 for Neck BSI (*p* = 0.02); 1.27 for Total Hip BSI (*p* = 0.004) and 1.35 for Spine BSI (*p* < 0.0001). After FRAX^®^ adjustment for the association continued to be statistically significant for Total Hip BSI (HR 1.24, *p* = 0.02 for all fragility fracture; 1.31, *p* = 0.01 for MOF) and Spine BSI (HR 1.33, *p* < 0.0001 for all fragility fracture; 1.33, *p* = 0.005 for major osteoporotic fracture; 1.67, *p* = 0.002 for clinical vertebral fracture) [55].

The capability of BSI to predict further vertebral fracture was also confirmed by Ulivieri et al. using an artificial intelligence analysis in a prospective, longitudinal, multicentric study of 172 female outpatients having at least one vertebral fracture at enrollment and who were monitored for an average period of 3 years. At the end of the follow-up, 93 women developed a further vertebral fracture, defined as one unit increase of SDI. Supervised Artificial Neural Networks (ANNs) analysis was used to distinguish women who developed another fracture from those who did not and to detect those variables providing the maximal amount of relevant information to discriminate between the two groups. ANNs choose appropriate input data automatically (TWIST-system, Training With Input Selection and Testing). Moreover, the authors built a semantic connectivity map using the Auto Contractive Map to provide further insights into the convoluted connections between the osteoporotic variables under consideration and the two scenarios (other fracture vs. no further fracture). TWIST system selected 5 out of 13 available variables: age, menopause age, BMI, FTot BMC, FTot BSI. With the training testing procedure, ANNs reached predictive accuracy of 79.36%, with a sensitivity of 75% and a specificity of 83.72%. The semantic connectivity map highlighted the role of lumbar spine BSI in predicting the risk of a further fracture, being the variable most inked to the occurrence of further fractures [56].

ANNs analysis with a predictive tool (TWIST system) was also able to demonstrate that femoral BSI plays a role in the prediction of the first vertebral fragility fracture. This was indicated in a recent retrospective study by Ulivieri et al. conducted on 174 postmenopausal women without vertebral fractures with a predictive tool (TWIST system), and with an average follow-up of 3 years. A semantic connectivity map was built to analyse the connections among variables within the groups. ANNs reached a predictive accuracy of 79.56% within the training testing procedure, with a sensitivity of 80.93% and a specificity of 78.18%. The semantic connectivity map showed that a low BSI at the total femur is connected to the absence of vertebral fragility fracture [26].

### 2.2. Secondary Osteoporosis

Lumbar BSI also permits the detection of patients affected by secondary osteoporosis in rare diseases, like those affecting young patients (Table 4).

Ulivieri et al. presented normative BSI data in a retrospective study of seventy haemophilic patients in different age decade classes from ≤30 to ≥71. Between the different age groups for BSI, *p*-value was = 0.0483 with no significant comparisons of the lowest age group versus the others [57], from a statistical point of view.

Rodari et al. investigated BSI in fifty children affected by neurofibromatosis type I. They found that pubertal patients showed significantly higher BSI mean values than prepubertal ones, but without statistically significant correlation with laboratory or densitometric data [58].

In a cohort of patients affected by mastocytosis [96 consecutive patients (46 women and 50 men) suffering from cutaneous or systemic mastocytosis], the authors found a correlation between high lumbar BSI and severity of bone deterioration. Tryptase presented an inverse correlation with lumbar BMD (r = −0.232; *p* = 0.022) and TBS (r = −0.280; *p* = 0.005), and directly with lumbar BSI (r = 0.276; *p* = 0.006). Lumbar BSI continued to be statistically significant (*p* = 0.006; adjusted R^2^ = 0.101) in the multivariate regression model with tryptase as a dependent variable. On the opposite side, lumbar BMD and TBS were statistically not significant. Tryptase increased by about 22 units for each unit increase of lumbar BSI. In addition, lumbar BSI resulted to be statistically significantly lower in women than in men, suggesting that men have a minor lumbar bone resistance to compressive loads, which is consistent with more severe bone involvement in mastocytosis in the males [27] (Table 1).

Tabacco et al. demonstrated that BSI is impaired in primary hyperparathyroidism, which may help to recognise patients at high fracture risk [59]. The authors studied 50 patients with primary hyperparathyroidism and 100 age- and sex-matched control subjects. They found that BSI was significantly higher at lumbar spine (2.28 ± 0.59 vs. 2.02 ± 0.43, *p* = 0.009), femoral neck (1.72 ± 0.41 vs. 1.49 ± 0.35, *p* = 0.001) and total femur (1.51 ± 0.33 vs. 1.36 ± 0.25, *p* = 0.002) in hyperparathyroid patients respect to controls. Lumbar spine BSI showed reasonable accuracy for discriminating vertebral fractures (AUC 0.667; 95% CI, 0.513–0.820) and lumbar spine BSI ≥ 2.2 was a statistically significant independent forecaster of vertebral fractures, with an adjusted odds ratio ranging from 5.7 to 15.1.

### 2.3. Therapy Monitoring

The ability of BSI to monitor the consequences and efficiency of anabolic treatment for osteoporosis has been assessed in a cohort of fractured osteoporotic women [25] (Table 5).

Forty fractured osteoporotic patients were studied before and after daily subcutaneous 20 mcg of teriparatide. BMD, HSA, TBS and BSI were measured and analysed, using a classical statistical approach and ANNs. The authors demonstrated significant improvements, after therapy, in BSI (−13.9%), TBS (5.08%), BMD (8.36%). Separating patients between responders and non-responders to the therapy, based on BMD increase >10%, the first group presented TBS and BSI improvements (11.87% and −25.46%, respectively). In comparison, the second group showed improvement in BSI only (−6.57%). This finding proposes that growth in bone strength that explains the well-known reduction in risk for fracture is not completely identified by an increase in BMD.

## 3. Conclusions

Osteoporosis is characterised by diminished bone quantity and, equally importantly, by reduced bone quality. For a complete evaluation of bone status, in addition to quantity of bone, information about its three-dimensional distribution, its geometry and its strength is required, as these elements contribute to skeletal resistance to load and fatigue. To better understand the risk of fracture in various conditions and to enable clinicians to properly manage osteoporotic patients, knowledge of all the involved factors in mechanical bone resistance is necessary. BSI is an index of bone strength that provides information about skeletal resistance to loads which is missing from existing indexes (BMD, HSA, TBS, HAL). The availability of this information improves the predictability of fragility fractures in osteoporotic patients. Further researches are underway to confirm the results obtained so far on broader case studies and in other secondary osteoporosis studies. TBS is a well-known index of bone texture, that discriminates fractured patients and that can foresee fractures in primary and secondary osteoporosis with bone architecture damage.

Even though femur size and shape are critical for the mechanical strength of the hip under various loading conditions and HAL shows promising results in fracture risk prediction in postmenopausal women, HSA needs further evidence to confirm its ability to discriminate fractured patients and to predict fragility fractures.

## Figures and Tables

**Table 1:** BSI experimental data. BV: bone volume. TV: trabecular volume. BSI: bone strain index. BMD: bone mineral density. TBS: trabecular bone score.

Topic	Author	Year	Specimen	Main Findings
Calculated strain and fatigue	Buccino et al.	2021	Porcine vertebra	Strain shows an increasing trend related to the number of cycles: ΔStrain Time1-Pre-fatigue = 42%; ΔStrain Time2-Pre-fatigue = 82%; ΔStrain Post-fatigue-Pre-fatigue = 91%, where the number of cycles Pre-fatigue < Time1 < Time2 < Post-fatigue Highest strain values correspond to lowest value of BV/TV. Damage occurs locally and starts in weaker regions and when further load is applied, it spreads to the other areas of the bone
Lumbar strain and mechanical static test	Colombo et al.	2019	Porcine vertebra	BSI showed a higher correlation with the ultimate stress, σ_ULT_ (R^2^_adj_ = −0.65) respect to Bone Mineral Density, BMD (R^2^_adj_ = 0.34) and Trabecular Bone Score, TBS (R^2^_adj_ = −0.03).

**Table 2:** BSI reproducibility data. BSI: bone strain index. BMD: bone mineral density. CoV: coefficient of variation. BMI: body mass index. HD: high definition.

Topic	Author	Year	Patients No.	Main Findings
BSI Hip reproducibility	Messina et al.	2020	30	BSI reproducibility was lower than that of BMD, confirming previous results of lumbar spine BSI. Total Femur reproducibility in vivo (CoV = 3.89%, reproducibility = 89.22%) was better in comparison to that of Femur Neck (CoV = 4.17%, reproducibility = 88.46%).
In Vivo Reproducibility	Messina et al.	2020	150	The group with BMI between 25 and 30 kg/m^2^ (CoV 1.97%, reproducibility 94.5%) showed the best reproducibility, while the worst was found in group with BMI > 30 kg/m^2^ (CoV 3.96%, reproducibility 89.0%). BSI reproducibility progressively worsened from lower BMI to higher BMI, but this reduction was not statistically significant.
In Vitro Reproducibility	Messina et al.	2019	Phantom based study	BSI reproducibility ranged from 98.3% (1-cm soft tissue thickness, HD-mode), to 96.1% (6 cm of superimposed soft tissue). Variations between scans at different superimposed tissue thicknesses were between 0.76% and 1.46% for BMD and between 1.03% and 1.57% for BSI.

**Table 3:** BSI clinical data: primary osteoporosis. BSI: bone strain index. FRAX: fracture risk assessment tool (Centre for Metabolic Bone Diseases, University of Sheffield, UK). HR: hazard ratio. MOF: major osteoporotic fracture. Fx: fracture. VFx: vertebral fracture. LBSI: lumbar bone strain index. VF: vertebral fracture. ANNs: artificial neural network analysis. BMD: bone mineral density. TBS: trabecular bone score.

Topic	Author	Year	Patients No.	Main Findings
Prediction of fragility fractures	Sornay-Rendu et al.	2022	846	BSI value was positively associated with a significant increase of the risk of all fragility Fx with an age-adjusted HR of 1.23 for Neck BSI (*p* = 0.02); 1.27 for Total Hip BSI (*p* = 0.004) and 1.35 for Spine BSI (*p* < 0.0001). After adjustment for FRAX^®^, the association remained statistically significant for Total Hip BSI (HR 1.24, *p* = 0.02 for all fragility Fx; 1.31, *p* = 0.01 for MOF) and Spine BSI (HR 1.33, *p* < 0.0001 for all fragility Fx; 1.33, *p* = 0.005 for MOF; 1.67, *p* = 0.002 for clinical VFx).
Prediction of fragility fracture (Artificial intelligence-based analysis)	Ulivieri et al.	2021	174	ANNs showed a predictive accuracy of 79.56% in the training test, a sensitivity of 80.93% and a specificity of 78.18%. The semantic connectivity map highlighted how low total femur BSI values are connected to the absence of VFs.
Prediction of vertebral refracture (Artificial intelligence-based analysis)	Ulivieri et al.	2021	172	ANN showed an accuracy of 79.36%, a sensitivity of 75% and a specificity of 83.72%. LBSI appeared to be the first bone variable directly related to the fracture event, indicating degrading in bone strength (LBSI high) as a significant risk factor for further VF.
Prediction of vertebral refracture (Multicentric Retrospective study)	Messina et al.	2020	234	BSI hazard ratio of incident re-fracture (95% CI) was 1.372 (1.038–1.813), *p* value = 0.0261, proportionality test *p* value: 0.5179.
Prediction of vertebral refracture (Retrospective study)	Ulivieri et al.	2020	143	The hazard ratio of refracture for each unit increase of BSI, BMD and TBS were respectively 1.201, 0.231 and 0.034. BSI resulted to be the parameter closest to the refracture event, with greater values associated to higher refracture risk.
Clinical observational retrospective study	Ulivieri et al.	2018	125	The semantic connectivity map showed that high lumbar BSI values, together with positive Romberg test, are connected to the fracture event. On the other hand, low carboxy-terminal cross-linking telopeptide of type I collagen level appeared to be related to low fracture risk.

**Table 4:** BSI clinical data: secondary osteoporosis. LS: lumbar spine. FN: femoral neck. TH: TH: femoral trocanther. PHPT: primary hyperparathyroidism. LS-BSI: lumbar spine bone strain index. VFs: vertebral fracture. AUC: area under the curve. BMD: bone mineral density. TBS: trabecular bone score. HSA: hip structural analysis. aBMD: areal bone mineral density. BMAD: bone mineral apparent density.

Topic	Author	Year	Patients No.	Main Findings
BSI in Hyperparathyroidism	Tabacco et al.	2021	150	BSI was significantly higher at LS (2.28 ± 0.59 vs. 2.02 ± 0.43, *p* = 0.009), FN (1.72 ± 0.41 vs. 1.49 ± 0.35, *p* = 0.001) and TH (1.51 ± 0.33 vs. 1.36 ± 0.25, *p* = 0.002) in PHPT. LS-BSI presented moderate accuracy for discriminating VFs (AUC 0.667; 95% CI 0.513–0.820). LS-BSI ≥ 2.2 and was an independent statistically significant predictor of VFs.
Bone Geometry and Structural Indexes in Mastocytosis (Retrospective Study)	Ulivieri et al.	2020	96	Tryptase presented an inverse correlation with Lumbar Spine BMD (r = −0.2326; *p* = 0.0226) and with TBS (r = −0.2801; *p* = 0.0057) and a direct correlation with Lumbar BSI (r = 0.2759; *p* = 0.0065). In the final multivariate regression, the Lumbar BSI in systemic Mastocytosis (*p* = 0.0064) and non-systemic Mastocytosis (*p* = 0.0338) maintained statistical significance.
DXA derived parameters in haemophilic patients (Retrospective study)	Ulivieri et al.	2018	70	Reduced bone mass was detected in 54.3% of the patients at lumbar spine, in 55.7% at femoral neck, and finally in 18.6% at total femur, Lumbar spine BMD, TBS and lumbar BSI were not correlated with HJHS (Haemophilia Joint Health Score). Bone geometry HSA parameters were negatively correlated with HJHS.
Neurofibromatosis type I	Rodari et al.	2018	125	Lumbar spine aBMD Z-score (r = −0.54, *p* < 0.0001), LS BMAD Z-score (r = −0.53, *p* < 0.0001), and TB Z-score (r = −0.39, *p* = 0.005) were negatively correlated with growth and pubertal development (*p* = 0.007, *p* =0.02, *p* = 0.01, respectively), indicating that patients did not gain as much as expected for their age. Bone strain did not show statistically significant correlation with any laboratory or densitometric data, although pubertal patients presented mean values significantly higher than prepubertal ones

**Table 5:** BSI monitoring osteoporosis therapy data. TBS: trabecular bone score. BMD: bone mineral density. HSA: hip structural analysis. FS_BMD: femoral shaft bone mineral density. FS_CSA: femoral shaft cross sectional area. FS_SEC_MOD: femoral shaft section modulus. FS_BR: femoral shaft buckling ratio.

Topic	Author	Year	Patients No.	Main Findings
DXA parameters response to teriparatide (Retrospective study)	Messina et al.	2020	40	In the entire population, the improvements post therapy regarded BSI (−13.9%), TBS (5.08%), BMD (8.36%). Significant HSA variations but of very small entity were present only at the femoral shaft (FS_BMD (0.23%), FS_CSA (−0.98%), FS_SEC_MOD (−2.33%) and FS_BR (1.62%)).

## Data Availability

Not applicable. All data cited are available as published work.
